# Definitive Chemoradiation for Unresectable Hyalinizing Clear Cell Carcinoma of the Base of the Tongue: A Molecularly Confirmed Case

**DOI:** 10.1155/crom/6717205

**Published:** 2025-12-15

**Authors:** Paul J. Pecorin, Mary Allen-Proctor, Samer Al-Khudari, Daniel W. Golden, Koosha Paydary

**Affiliations:** ^1^ Department of Medicine, Rush University Medical Center, Chicago, Illinois, USA, rush.edu; ^2^ Department of Pathology, Rush University Medical Center, Chicago, Illinois, USA, rush.edu; ^3^ Department of Otorhinolaryngology-Head and Neck Surgery, Rush University Medical Center, Chicago, Illinois, USA, rush.edu; ^4^ Department of Radiation Oncology, Rush University Medical Center, Chicago, Illinois, USA, rush.edu; ^5^ Division of Hematology, Medical Oncology and Cell Therapy, Department of Medicine, Rush University Medical Center, Chicago, Illinois, USA, rush.edu

## Abstract

Hyalinizing clear cell carcinoma (HCCC) is a rare malignancy of the minor salivary glands, most often managed by surgical resection. We report a case of a 63‐year‐old woman with an unresectable base‐of‐tongue tumor initially presumed to be squamous cell carcinoma. Histopathologic evaluation and molecular testing ultimately confirmed HCCC with an EWSR1‐ATF1 fusion. Given the tumor’s extent, she was treated with definitive chemoradiation using weekly cisplatin and 70 Gy in 35 fractions. Her course was complicated by pulmonary embolism, neutropenia, and severe mucositis requiring percutaneous endoscopic gastrostomy tube placement. Post‐treatment imaging showed decreased FDG avidity, and circulating tumor DNA remained negative for minimal residual disease. This case highlights the importance of molecular diagnostics in distinguishing HCCC from other clear cell neoplasms and suggests a potential role for chemoradiation in unresectable cases, though treatment‐related toxicity remains a significant concern. Further investigation into systemic and targeted therapies for HCCC is warranted.

## 1. Introduction

Salivary gland cancers represent a rare and histologically diverse group of malignancies that arise in the parotid, submandibular, or minor salivary glands, accounting for approximately 5% of all head and neck tumors [1]. Among these, hyalinizing clear cell carcinoma (HCCC) is a rare subtype, characterized by clear cell nests separated by hyalinizing stroma and the presence of the EWSR‐ATF1 fusion gene [2]. Because of its rarity, there is limited data on optimal management strategies and long‐term outcomes.

Typically, the standard treatment for HCCC involves surgical resection, either alone or followed by adjuvant radiation therapy. There is a paucity of data regarding the role and efficacy of chemotherapy, as only a small group of patients received chemotherapy, ranging from 1.3% in one literature review [2] to 4.1% in one retrospective cohort study [3]. Additionally, multiple case reports report the use of chemotherapy in more advanced cases of HCCC [4, 5]. We present a case of a patient with HCCC originating from the base of the tongue (BOT), which was not amenable to surgical resection. The patient was treated with definitive chemoradiation therapy using cisplatin, providing insight into this rare clinical scenario.

## 2. Case

### 2.1. Initial Evaluation

A 63‐year‐old female presented to the emergency department with persistent sinus pressure and ear pain following a COVID‐19 infection. Her medical history was notable for chronic, intermittent sinus congestion, ear pain, and throat burning. A CT of the neck demonstrated an ulcerated mass measuring 3.5 × 1.8 × 3.2 cm in the midline base of tongue, with invasion of the right genioglossus musculature and extension into the pre‐epiglottic space, sparing the fat. Additionally, a right‐sided level IIA lymph node measuring 1.5 × 1.2 cm was identified. These findings were suspicious for primary oropharyngeal squamous cell carcinoma (SCC).

### 2.2. Diagnostic Workup

**Figure 1:**
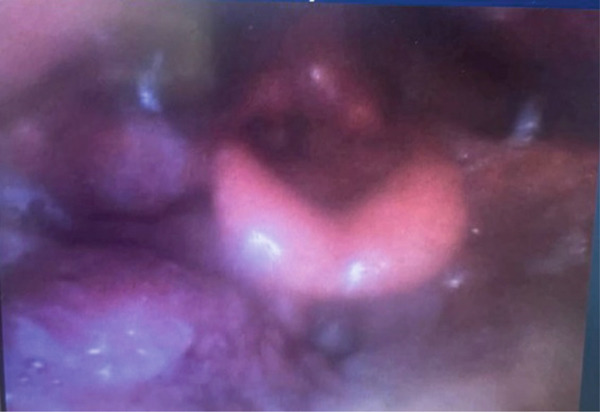
Laryngoscopy photo on initial presentation following biopsy at outside facility demonstrating post‐biopsy changes of the base of tongue lesion and prominent lingual tonsils.

**Figure 3:**
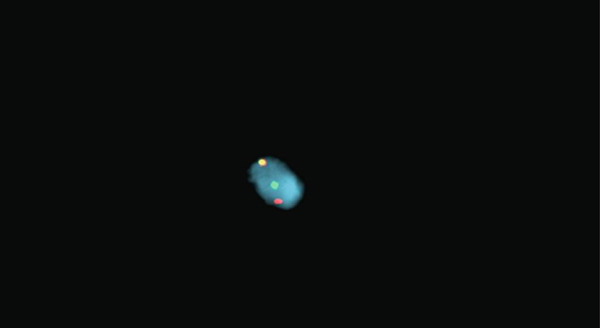
Detection of EWSR1 gene rearrangement in hyalinizing clear cell carcinoma. Fluorescence in situ hybridization (FISH) using a dual‐color break‐apart probe targeting the *EWSR1* gene (22q12) was performed. A normal fused signal (yellow) indicates no rearrangement, whereas split red and green signals indicate a rearrangement. Among 200 nuclei analyzed, 177 (88.5%) demonstrated break‐apart signals, confirming the presence of an *EWSR1* gene rearrangement.

**Figure 4:**
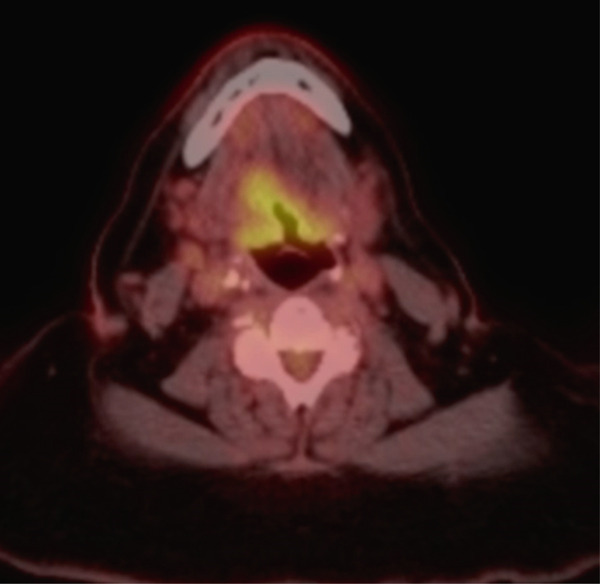
Pretreatment PET/CT demonstrates a large, lobulated, hypermetabolic lesion involving the posterior tongue with central ulceration, concerning for malignancy, along with FDG‐avid right level 1B and II cervical lymph nodes consistent with metastatic disease.

The patient underwent microlaryngoscopy and biopsy of the mass (Figure 1). Initial histopathological findings suggested moderately differentiated SCC, with tumor cells testing positive for cytokeratin 5/6 and p40, but negative for p16 (Figure 2). However, fluorescence in situ hybridization (FISH) targeting the EWSR1‐ATF1 gene rearrangement showed 177 of 200 analyzed nuclei (88.5%) positive for the EWSR1 gene rearrangement, more consistent with HCCC (Figure 3). Next generation sequencing showed a PDL1 combined positive score of 1, a tumor mutational burden of 1.1 mutations/Mb, stable MSI, a STAG‐2 mutation with 10.6% variant allele frequency and confirmed an EWSR‐ATF1 rearrangement. Subsequent PET‐CT revealed a hypermetabolic soft tissue lesion along the posterior tongue corresponding to the known HCCC. FDG‐avid right level IB and II neck nodes were consistent with metastases, while mildly FDG‐avid left level II nodes suggested possible metastases (Figure 4). The patient was diagnosed with a HCCC, cT4aN2M0, Stage IVA.

Figure 2Hematoxylin and eosin stains from the base of tongue biopsy shown at 10× (a) and 4× (b–f). Outside hospital immunohistochemistry showed tumor cells positive for CK5/6 and p40, and negative for p16. This was initially thought to be consistent with moderately differentiated invasive squamous cell carcinoma.

**Figure:**
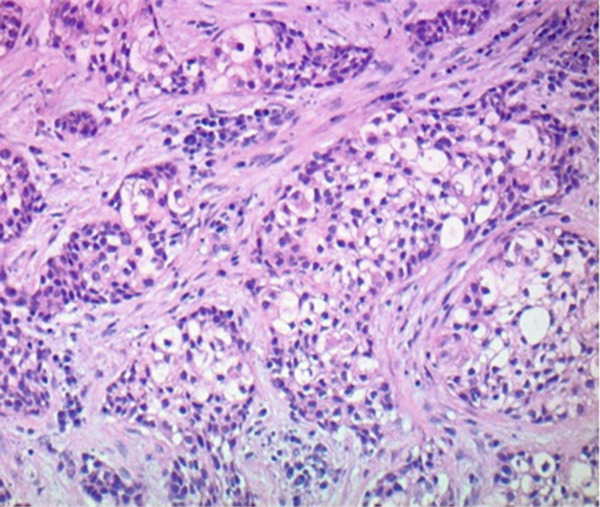
(a)

**Figure:**
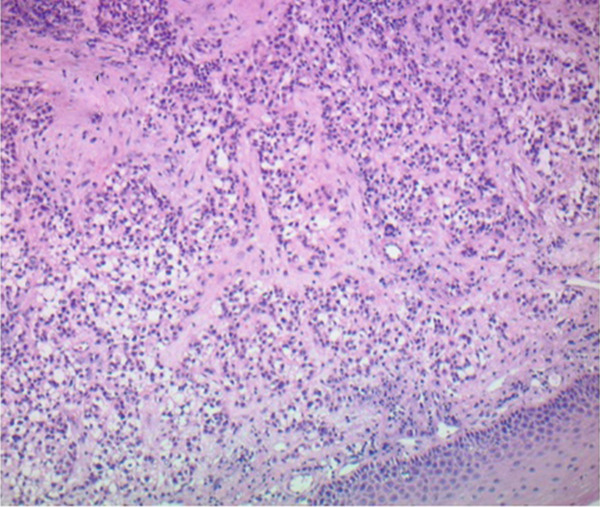
(b)

**Figure:**
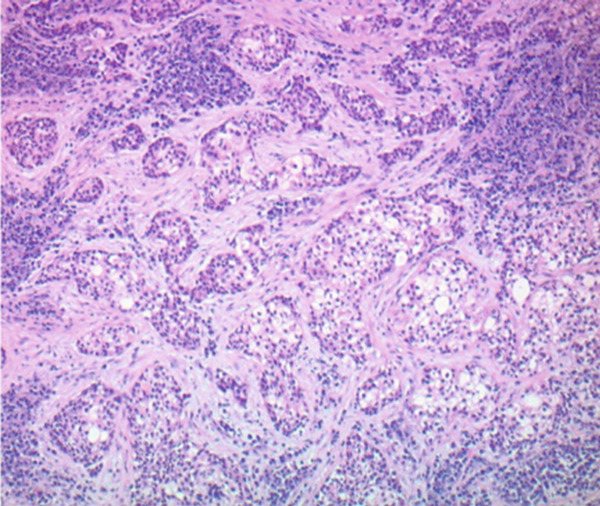
(c)

**Figure:**
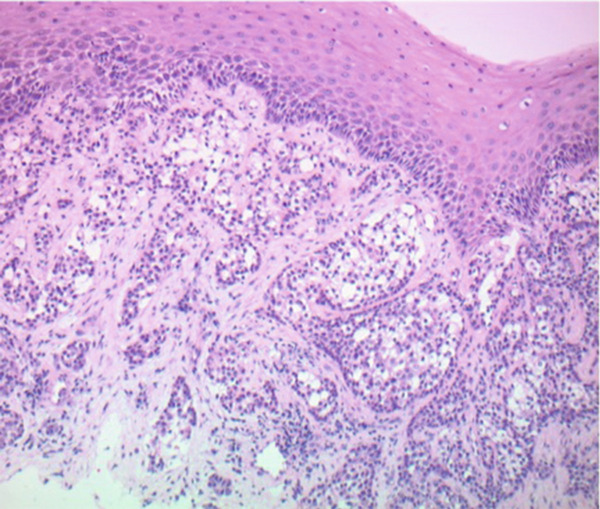
(d)

**Figure:**
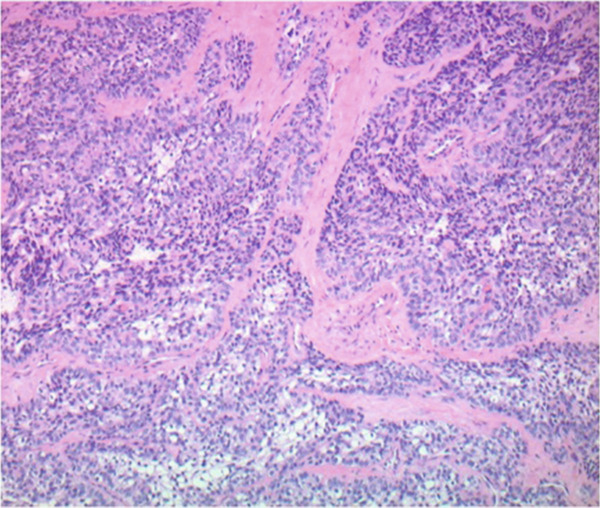
(e)

**Figure:**
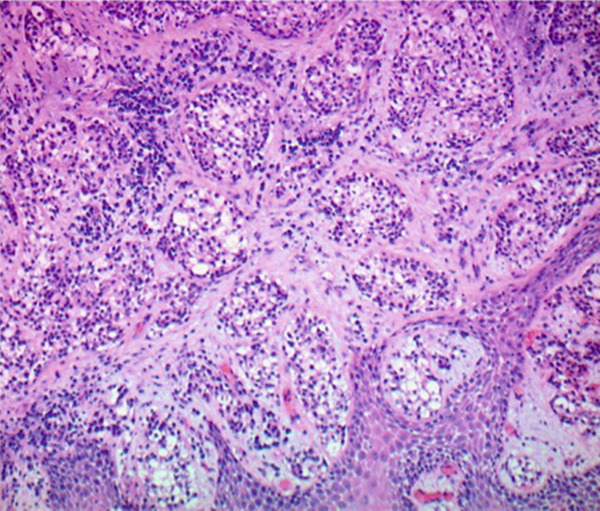
(f)

### 2.3. Treatment Course

Although surgical resection is considered the standard of care for HCCC, it was deemed to not be feasible because of tumor location and extent. The patient began chemoradiation therapy, receiving weekly cisplatin (40 mg/m^2^) with concurrent radiation therapy (70 Gy in 35 fractions) targeting the BOT and bilateral neck. Her treatment course was complicated by pulmonary embolism, neutropenia, and severe mucositis, necessitating hospitalization and placement of a percutaneous endoscopic gastrostomy (PEG) tube. Because of her hospitalization, she did not complete the planned seven cycles of weekly cisplatin, only receiving four cycles.

### 2.4. Outcomes

**Figure 5:**
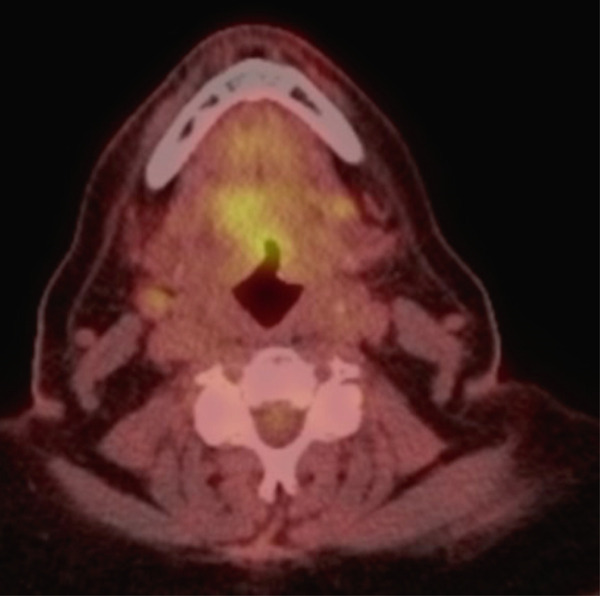
Post‐treatment PET/CT demonstrates a persistent right base‐of‐tongue mass with central ulceration and interval decreased FDG avidity, likely reflecting a combination of treated residual disease and postradiation changes. There is also mild interval decrease in size of a right level II lymph node with relatively stable FDG uptake and no new lymphadenopathy. Because of timing of imaging, findings felt to be most consistent with postradiation change rather than residual disease.

**Figure 6:**
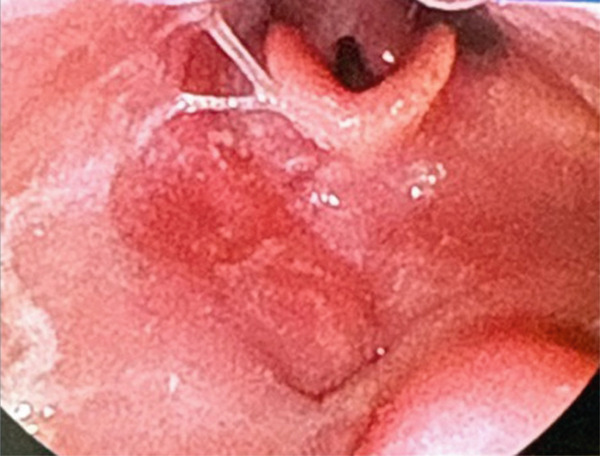
Laryngoscopy photo 10 months after completing chemoradiation therapy showing resolution of base of tongue mass and improvement of post treatment changes.

About 3 months after completing the course of treatment, repeat PET‐CT demonstrated reduced FDG avidity in the primary lesion and right level II lymph nodes. Residual FDG avidity was attributed to postradiation changes versus residual disease (Figure 5). Biopsy via laryngoscopy was considered but deferred because of delayed mucosal healing and lack of a clear target. Given the timing, the FDG avidity was felt more likely to represent postradiation inflammation rather than persistent tumor and proceeding with biopsy risked poor healing and unnecessary morbidity. The patient experienced gradual improvement in mucositis and odynophagia, leading to removal of the PEG tube. Follow‐up CT scans showed stable to marginally decreased size of the BOT lesion and reduced size of the cystic metastatic right level II lymph node. One year after treatment, laryngoscopy showed resolution of the BOT lesion (Figure 6). Circulating tumor DNA testing for evaluation of minimal residual disease (MRD) has remained negative. The patient continues to be monitored with periodic staging CT scans and MRD testing every 3 months.

A timeline of events can be seen in Table 1.

**Table 1:** Timeline of events utilizing initial CT scan with concern for malignancy as Day 1.

**Interval from Day 1**	**Event**
Day 1	CT neck revealed a 3.5 × 1.8 × 3.2 cm ulcerated base‐of‐tongue mass with right genioglossus invasion and a right level IIA lymph node, suspicious for malignancy
Day 21	Biopsy confirmed hyalinizing clear cell carcinoma (HCCC) via histopathology and EWSR1‐ATF1 rearrangement testing
Day 55–Day 111	Definitive chemoradiation initiated with weekly cisplatin (planned 7 cycles, completed 4) and concurrent 70 Gy in 35 fractions; course complicated by pulmonary embolism, neutropenia, and severe mucositis requiring hospitalization and PEG placement
Day 197 (≈3 months post‐treatment)	PET‐CT demonstrated reduced FDG avidity in the primary lesion and right level II lymph nodes, likely postradiation changes; biopsy deferred because of delayed mucosal healing and lack of clear target
Day 228	PEG tube removed following gradual improvement in mucositis and odynophagia
Day 279 (≈6 months post‐treatment)	Follow‐up CT showed stable to marginally decreased size of the BOT lesion and reduced size of the cystic metastatic right level II lymph node
Day 455 (≈12 months post‐treatment)	Laryngoscopy demonstrated resolution of the BOT lesion; circulating tumor DNA for MRD remained negative

## 3. Discussion

HCCC is a rare tumor of the oropharynx arising from the minor salivary glands, accounting for only 2.5% of malignant salivary gland tumors [6]. Because of histologic overlap with other clear cell neoplasms, such as clear cell SCC and mucoepidermoid carcinoma, HCCC has posed diagnostic challenges. In our patient, initial histopathologic findings were positive for cytokeratin 5/6 and p40 staining with negative staining for p16. This was initially thought to be consistent with SCC, however, EWSR1‐ATF1 rearrangement was identified, which confirmed the diagnosis of HCCC. This highlights the importance of molecular diagnostics in the identification of HCCC [2, 3].

In most cases, HCCC is treated with surgical resection combined with adjuvant radiation therapy. Only a small proportion of patients receive chemotherapy, with studies suggesting between 1% and 9% receiving chemotherapy [2, 3, 7, 8]. Given the paucity of data, there are no recommended chemotherapy guidelines for HCC, but prior cases have demonstrated the efficacy of platinum‐based chemotherapies such as cisplatin and carboplatin with or without paclitaxel [4, 5]. Specifically, there have been cases demonstrating complete response to cisplatin based chemotherapy in a recurrent HCC [4] as well as two cases of HCCC treated with paclitaxel and carboplatin with no evidence of recurrence at follow‐up [5]. Despite case reports demonstrating efficacy, one retrospective cohort study indicates that patients receiving chemoradiation therapy along with surgery had an increased risk of 3‐year mortality as compared with surgery alone with a hazard ratio of 3.69 [3]. This is likely due to increased disease severity in those receiving chemoradiation. In our case, chemoradiation with cisplatin was utilized because of the tumor’s location and extent. Although HCCC tumors are typically chemo‐resistant, cisplatin was chosen for radio‐sensitization, aligning with treatments for other head and neck tumors as well as prior case reports [3–5]. The patient experienced significant treatment‐related toxicities including pulmonary embolism, neutropenia, and severe mucositis requiring PEG tube placement. Therefore, she was only able to receive four cycles of the weekly cisplatin. Despite this, there was reduced FDG avidity on post‐treatment PET‐CT and negative circulating tumor DNA indicative of a favorable response.

Although PET‐CT and exam indicate a positive response, the inability to verify treatment response histologically is a significant limitation of this case. Biopsy was deferred because of delayed mucosal healing and lack of a clear target, as a result, residual microscopic disease could not be definitively excluded. This limitation underscores the challenge of accurately assessing treatment response in unresectable head and neck cancers when biopsy poses significant morbidity. This case is also limited by short term follow‐up. This patient will need continued monitoring for recurrence going forward.

The significant toxicity associated with chemoradiation for unresectable HCCC underscores the need for novel therapeutic approaches, including targeted and epigenetic therapies. The EWSR1–ATF1 fusion gene seen in HCCC also occurs in other malignancies, such as angiomatoid fibrous histiocytoma, myoepithelial tumors, and clear cell sarcoma (CSS) [9]. In CSS, preclinical mechanistic studies have shown that histone deacetylase (HDAC) inhibitors such as vorinostat can downregulate EWSR1–ATF1 expression, in part through reduced promoter occupancy by BRD4, a member of the bromodomain and extra‐terminal (BET) family [10]. Combination therapy with vorinostat and BET inhibitors has demonstrated synergistic suppression of the fusion gene and enhanced antiproliferative activity in vitro, although the precise mechanisms remain under investigation [10]. To our knowledge, neither HDAC inhibitors nor BET inhibitors have been evaluated specifically in HCCC. However, several BET inhibitors are currently being investigated in early‐phase clinical trials for advanced solid tumors [11].

This case emphasizes the limited research on HCCC, especially in unresectable cases. Further studies on the efficacy of chemoradiation are warranted, though the feasibility of large‐scale trials remains questionable. Targeted therapies for the EWSR1‐ATF1 fusion gene could be a future research direction given current data specific to HCCC are lacking.

## 4. Conclusion

HCCC of the oropharynx presents a unique diagnostic and therapeutic challenge. This case highlights the importance of molecular diagnostics, particularly in tumors with clear cell features or ambiguous head and neck pathology, as identification of the EWSR‐ATF1 fusion directly influences diagnosis and may impact management. For unresectable disease, chemoradiation may offer meaningful tumor control, although treatment‐related toxicities can be substantial, highlighting the need for careful patient selection and supportive care.

Given the scarcity of data on nonsurgical management of HCCC, individual case reports such as this are important contributions to collective clinical understanding. Future efforts should include systematic reporting of similar cases, incorporation into multicenter registries, and exploration of targeted therapies directed at the EWSR1–ATF1 fusion. Continued accumulation of such cases may eventually guide consensus recommendations for managing unresectable presentations of this rare tumor.

## Consent

No written consent has been obtained from the patients as there is no patient identifiable data included in this case report.

## Disclosure

The authors have nothing to report.

## Conflicts of Interest

The authors declare no conflicts of interest.

## Funding

No funding was received for this manuscript.

## Data Availability

Data sharing not applicable to this article as no datasets were generated or analyzed during the current study.
